# Suitable carrier protein and linker peptide significantly increase the secretory expression of human lysozyme in *Aspergillus niger*: Suitable carrier protein and linker peptide of *A*. *niger*

**DOI:** 10.3724/abbs.2023153

**Published:** 2023-09-04

**Authors:** Qi Wu, Can Xu, Wei Shi, Lifang Li, Hui Zhang, Tianqi Liu, Junbo Fan, Lingmeng Cui, Jie Li

**Affiliations:** College of Life Sciences Northeast Agricultural University Harbin 150030 China

Lysozyme (LYZ) is an oval-shaped antimicrobial agent with good bacterial lytic properties and is widely distributed in human, animal, plant, and even some microbial tissues. It can destroy the walls of bacterial cells by cleaving the chemical bond linking N-acetylglucosamine and N-acetyl carbamate in the peptidoglycan layer. Due to its superior bactericidal and anti-inflammatory properties, human LYZ (hLYZ) is used as an alternative to antibiotics, food additive, feed additive, and anti-infection agent [1]. Currently, hLYZ is mainly extracted from breast milk and the placenta, making its raw material scarce and costly, so its large-scale production has great limitation. Furthermore, since hLYZ is a eukaryotic protein containing four intramolecular disulfide bonds, its expression in *Escherichia coli* is not favored. This suggests that eukaryotic cells with more complex expression systems are a better host choice for hLYZ production [2].

*Aspergillus niger* is one of the most widely used eukaryotic expression systems in biotechnology. It is a highly safe and food-grade strain with a short fermentation cycle, complete protein synthesis and modification system, and excellent protein secretion ability [3]. However, there is still a difference between the yield of exogenous protein and that of endogenous protein, even 10 ^2^ to 10 ^3^ times [4]. With the development of technology, a variety of genetic engineering methods and strategies have been used to improve the efficient expression of foreign proteins in filamentous fungi. The fusion protein strategy based on the sequential fusion of linker peptides is an important means to overcome the problem of low expression of foreign proteins. This strategy can reduce the degradation of heterologous proteins by the host system to a certain extent and is widely used with the highest success rate [5]. The linker peptide can also improve the folding and stability of fusion proteins and increase the expression of fusion proteins [6].

Currently, there are few fusion proteins suitable for the *A*. *niger* expression system, so exploring more fusion proteins ideal for the A. niger expression system is highly innovative. In this study, we selected five types of highly expressed endogenous proteins, including endoglucanase (EglA), endo-1,4-beta-xylanase (XynB), feruloyl esterase (FaeA), tannase (Tan), and glucose oxidase (GOD), of *A*. *niger* as carrier proteins and five different linker peptides, including (GGGGS) _1‒3_, (EAAAK) _2_, and (AP) _5_. The efficient expression of hLYZ in *A*. *niger* was taken as the research object to develop more fusion expression strategies suitable for the *A*. *niger* expression system to achieve the efficient expression of heterologous proteins in this system.

First, we successfully constructed recombinant expression vectors containing different carrier proteins and linker peptides using PCR amplification and pSZHG6R vectors stored in our laboratory. Second, the recombinant expression vector was transferred into AGL1 by the freeze-thaw method, and the AGL1-positive transformants were screened by PCR using primers P15 and P16 ( Supplementary Table S1 and Supplementary Figure S1). Subsequently, AGL1-positive transformants were cocultured with *A*. *niger* TH-2 ( Figure 1A). According to the principle of homologous recombination, the *glaA* gene locus in *A*. *niger* was replaced by a gene expression construct in the expression vector, and then homozygous recombinant strains were rescreened by PCR ( Supplementary Figure S2). Recombinant strains with different carrier proteins EglA, XynB, FaeA, Tan, and GOD were named ELF, XLF, FLF, TLF, and GLF, respectively. All of these recombinant strains used GGGGS as the linker peptide. The recombinant control strain without carrier protein was named LF.

**Figure 1:**
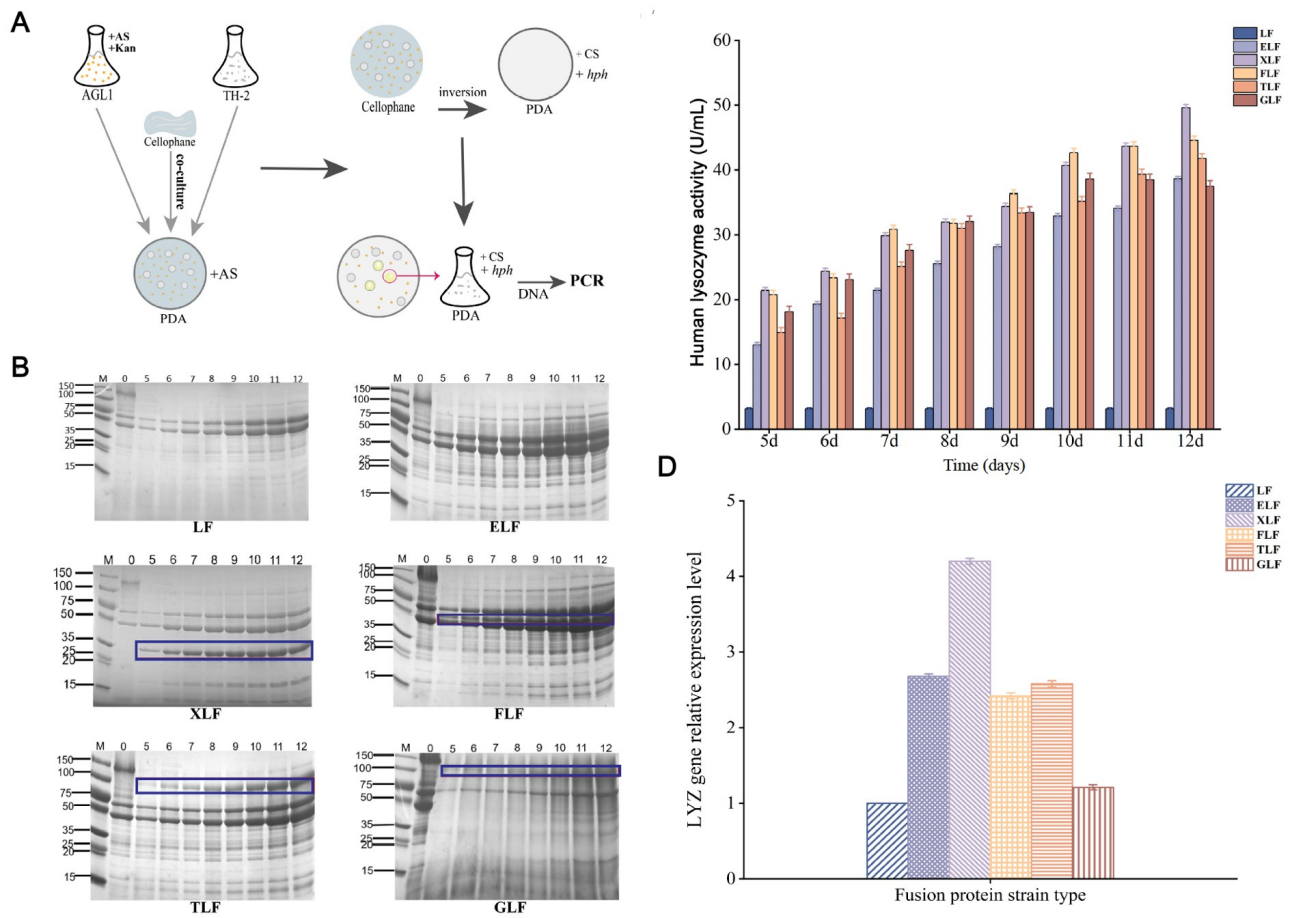
Figure 1 Construction and detection of recombinant strains of *A*. *niger* with different carrier proteins (A) Construction of recombinant strains of A. niger. (B) SDS-PAGE detection. Line 0 represents the starting strain, and Lines 5‒12 represent the number of days of fermentation; and the dark blue box is the fusion protein secreted by the recombinant strain. (C) Enzyme activity of hLYZ. (D) The transcription level of the target gene hLYZ.

Recombinant strains were fermented by shaking the flask. SDS-PAGE and the hLYZ activity (turbidimetric method) of the supernatant and the *hLYZ* gene transcription level were determined. The SDS-PAGE results showed that compared with the original strain TH-2, new fusion protein bands were found in the recombinant strains XLF, FLF, TLF, and GLF, but no new protein bands were found in the recombinant strains LF and ELF ( Figure 1B). This indicated that the highly expressed endogenous protein of *A*. *niger* as a carrier protein could effectively improve the expression level of hLYZ in *A*. *niger*. The amount of fusion protein secreted by XLF was significantly higher than that secreted by the other recombinant strains. This may be because XynB has the advantages of smaller molecular weight, higher hydrophilicity, and better ability to help fusion proteins secrete from intracellular to extracellular space. Regarding enzyme activity, except strain LF, strains ELF, XLF, FLF, TLF, and GLF increased from the 5th day and reached a maximum on the 12th day. The enzyme activities were 38.64 U/mL, 47.9 U/mL, 44.6 U/mL, 41.8 U/mL, and 37.5 U/mL, respectively ( Figure 1C). Therefore, it can be determined that the carrier protein can enhance the secretory expression of hLYZ in *A*. *niger*. However, the enzyme activity of the recombinant protein is not high, which may be due to the short flexible linker peptide GGGGS, resulting in the carrier protein affecting the spatial structure of hLYZ.

The samples on day 5 of fermentation were precipitated for RNA extraction and reverse transcribed to cDNA using a kit (CWBIO, Taizhou, China). Then, real-time quantitative PCR was performed. The results showed that the transcription levels of the *hLYZ* gene in ELF, XLF, FLF, TLF, and GLF were 2.68 times, 4.20 times, 2.42 times, 2.58 times, and 1.21 times as high as that of strain LF, respectively ( Figure 1D). This once again proved that the carrier protein could significantly improve the transcription level of the target gene *hLYZ*. However, the transcript level in strain ELF was significantly increased with some enzymatic activity, but no significant new fusion protein bands were detected. The reason may be that EglA, as a carrier protein, contributes little to the secretion of LYZ, and only a few fusion proteins were successfully transported out of the cells. However, for the linker peptide, the GGGGS may be broken, leaving the LYZ in an independent state, or the LYZ in the fusion protein can fold correctly, thus having high activity. The specific mode of action and changes in the fusion protein in ELF need to be further investigated and analyzed. In summary, xylanase XynB has significant advantages in protein expression, enzyme activity, and transcription levels, which makes it the best choice for a carrier protein. On this basis, the influence of linker peptides on the expression of *hLYZ* in *A*. *niger* was further explored.

Five recombinant strains with different linker peptides, including (GGGGS) _1‒3_, (EAAAK) _2_, and (AP) _5_ between XynB and hLYZ, were constructed and named XLF, XG _2_LF, XG _3_LF, XE _2_LF and XALF, respectively. SDS-PAGE and enzyme activity results of recombinant strains with different linker peptides showed that compared with the original strain TH-2, the protein band of hLYZ was approximately 14.7 kDa, and the fusion protein band was approximately 35 kDa. More importantly, we found that compared with strain XLF, some hLYZ in other recombinant strains could be secreted independently, and the linker peptide in the fusion protein could be broken ( Figure 2A). At the whole level, the hLYZ activity of strain XG _3_LF was highest (546.58 U/mL), and the independent expression of hLYZ in strain XE _2_LF was the best among all recombinant strains. This may be because the secondary structure of the flexible linker peptide (GGGGS) _n_ is a mainly random curly state, which allows the carrier protein to freely regulate the distance between the target protein and the carrier protein, thereby promoting the correct folding and secretion of hLYZ [7]. However, the rigid linker peptide (EAAAK) _n_ has a hydrogen bond and a tightly arranged main chain, and the secondary structure is an α-helix structure, so the structure is more rigid and stable. The rigid linker peptide (EAAAK) _2_ can maintain a stable distance between the carrier protein and the target protein while building fusion proteins without affecting the independent function [8]. pH value has also been shown to influence the salt bridge forces that maintain the stability of the rigid linker peptide EAAAK α-helix secondary structure. When the pH is neutral, it will lead to self-splitting of EAAAK [9]. This may be the main reason for the independent expression of the fusion protein in strain XE _2_LF.

**Figure 2:**
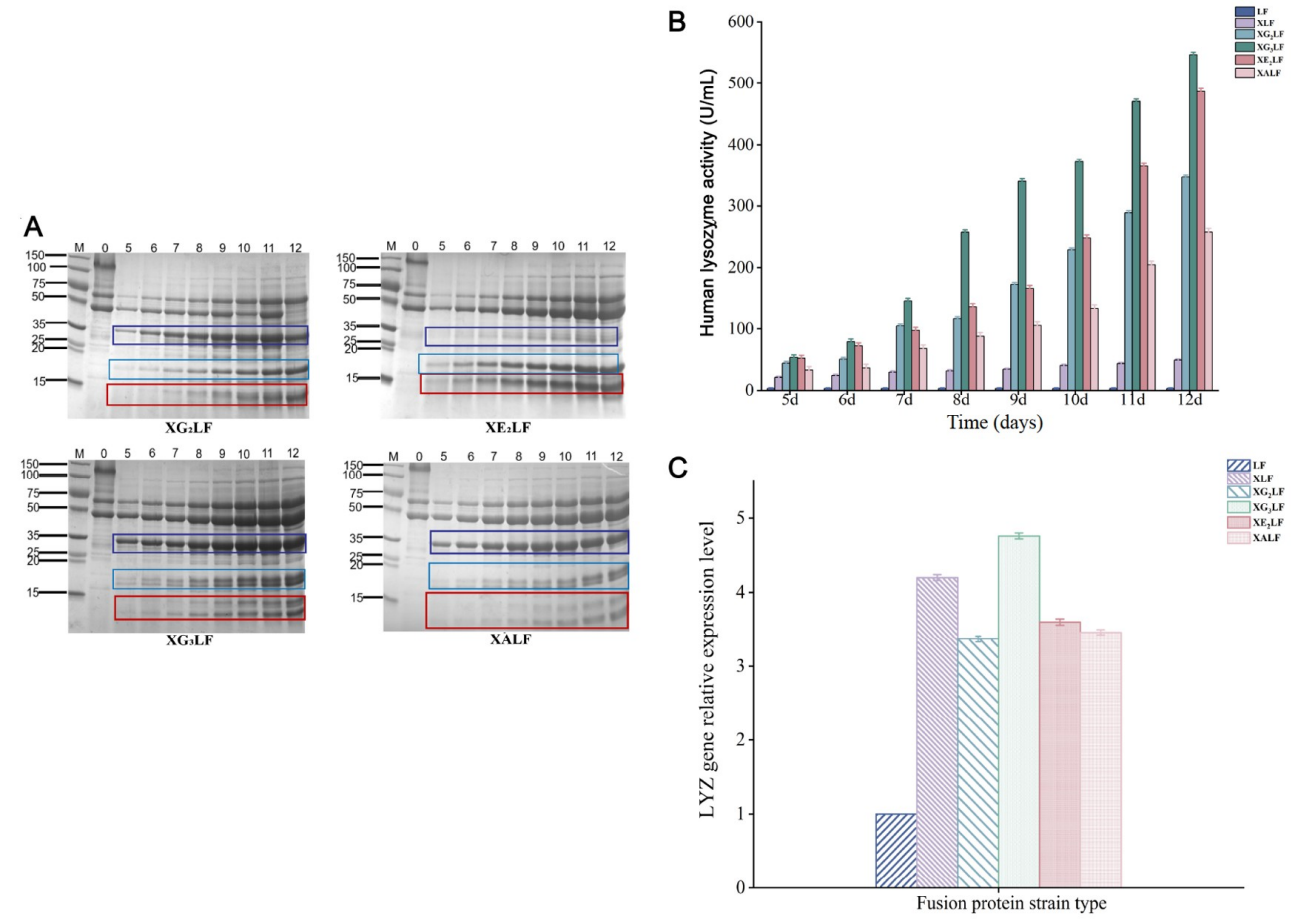
Figure 2 Detection of recombinant strains of *A*. *niger* with different linker peptides (A) SDS-PAGE detection. The dark blue box represents the fusion protein, the light blue box represents XynB, and the red box represents LYZ. The results of strain XLF are shown in Figure 1B. (B) Enzyme activity of hLYZ. (C) The transcriptional level of the target gene hLYZ.

Furthermore, we compared the enzyme activity of the recombinant strains containing flexible linker peptides and found that the order from high to low was XG _3_ LF>XG _2_LF>XLF (546.58 U/mL>346.67 U/mL>46.58 U/mL) ( Figure 2B). This indicated that the more copies of the flexible linker peptide, the more favorable the secretory expression of hLYZ. However, the protein-independent expression ability and enzyme activity (257.49 U/mL) of strain XALF were significantly lower than those of strain XE _2_LF (486.62 U/mL). The reason may be that the rigid linker peptide (XP) _n_ contains a proline preference sequence, which can increase the rigidity of the linker peptide, makes it difficult to break, and is not conducive to protein interaction and expression [10].

The transcription levels of the recombinant strains with different linker peptides showed that the transcription levels of the target gene *hLYZ* increased by 4.20 times, 3.37 times, 4.76 times, 3.60 times, and 3.46 times, respectively ( Figure 2C), compared with that of strain LF, indicating that the multiple changes among the strains are not significant. However, the change in the transcription level was consistent with the trend of protein expression and enzyme activity. Based on the above analyses, we found that strain XG _3_LF had the highest enzyme activity, indicating that the flexible linker peptide (GGGGS) _3_ has the best effect. The rigid linker peptide (EAAAK) _2_ was more effective in the independent expression of fusion proteins. Since the difference in enzyme activity between XG _3_LF and XE _2_LF is only 11%, both linker peptides can be selected.

In conclusion, a recombinant strain of *A*. *niger* expressing hLYZ was successfully constructed in this study, which provided a safe and efficient production mode for hLYZ. More importantly, this study explored the possibility of more carrier proteins and linker peptides suitable for the *A* . *niger* expression system. This provided strong support for the ability of the *A*. *niger* system to express more foreign proteins.

## Supplementary Data

Supplementary data is available at *Acta Biochimica et Biophysica Sinica* online.

## Supporting information

23122Supplementary_Data
