# Supplementary material for: Suitable carrier protein and linker peptide significantly increase the secretory expression of human lysozyme in Aspergillus niger : Suitable carrier protein and linker peptide of A. niger
Source: Acta Biochim Biophys Sin (Shanghai). 2023 Sep 4;55(10):1677–80. doi: 10.3724/abbs.2023153 (PMC10577450; doi:10.3724/abbs.2023153)
Supplement: 23122Supplementary_Data [file 23122Supplementary_Data.pdf]

Supplementary Table S1. Sequences of primers used in this study

Primer	Sequence (5'→3')	Purpose
P1: 5'*eglA*-sence	TCTAGATCTCAATATGACAGTGCCTCG	Clone *eglA* gene
P2: 3'*eglA*-antisence	AAGCTTGGATCCGCTAGCTCTCGGAGGAGGTGGG TTGACACTGGCGGTCC	Clone *eglA* gene
P3: 5'*xynB*-sence	TCTAGACACATCAAGCTCTCCC	Clone *xynB* gene
P4: 3'*faeA*-sence	TCTAGAGCCTCCACGCAAGGCATCTC	Clone *faeA* gene
P5: 5'*faeA*-antisence	AAGCTTGGATCCGCTAGCAGACCCTCCGCCTCC	Clone *faeA* gene
P6: 3'*Tan*-sence	TCTAGAGCAACTCCCTCCACGTTG	Clone *Tan* gene
P7: 5'*Tan*-antisence	AAGCTTGGATCCGCTAGCGGAGCCACCGCCACCG TACACAGGCATAGGAAC	Clone *Tan* gene
P8: 3'*GOD*-sence	GCTAGCAATGGCATTGAAGCCAG	Clone *GOD* gene
P9: 5'*GOD*-antisence	AAGCTTGGATCCGCTAGCAGACCCTCCGCCTCCC TGCATGGAAGCATAATC	Clone *GOD* gene
P10: 5'*xynB*G1-antisence	AAGCTTGGATCCGCTAGCAGACCCTCCGCCTCC	Add $(GGGGS)_1$
P11: 5'*xynB*G2-antisence	AAGCTTGGATCCGCTAGCGGAGCCAC CGCCACCGGAGCCACCGCCACCCT	Add $(GGGGS)_2$
P12: 5'*xynB*G3-antisence	AAGCTTGGATCCGCTAGCGGAGCCACCGCCACCG GAGCCACCGCCACCGGAGCCACCGCCACCCT	Add $(GGGGS)_3$
P13: 5'*xynB*E2-antisence	AAGCTTGGATCCGCTAGCCTTCGCAGCGGCCTCC TTCGCAGCGGCCTCCTGAACAGTGATGGACG	Add $(EAAAK)_2$
P14: 5'*xynB*P5-antisence	AAGCTTGGATCCGCTAGCGGCAGGGGCAGGTGC GGGCGCTGGAGCCGGCTGAACAGTGATGGACG	Add $(AP)_5$
P15: 3'gla*A*-sence	CGCTATAGGCTGGTTCTCC	Transformant identification
P16: 5'gla*A*-antisence	TCGGCTATTATTGAACTGGG	Transformant identification
P17: *bipA*-RT-sense	ACCAATACGCCGCCAACC	RT-qPCR
P18: *bipA*-RT-tisense	CAGCGTGGGTAACCTTCTTTC	RT-qPCR
P19: *pdiA*-RT-sense	AGCTCAAGGCGAAGAATATCCCT	RT-qPCR
P20: *pdiA*-RT-antisense	TAAGCTTGCTAGAGTCAACACC	RT-qPCR
P21: hacA-RT-sense	CGTCGGCTCAAACCCTGAACCTC	RT-qPCR
P22: hacA-RT-antisense	CTCCTCCGTCGAGAACGTCAAAGGC	RT-qPCR
P23: actA-RT-sense	CCACGAGACCACCTTCAACTCCA	RT-qPCR
P24: actA-RT-antisense	CCACCGATCCAGACGGAGTACTTGC	RT-qPCR
P25: *LYZ*-RT-sense	AATGATGGCAAAACCCCAGGA	RT-qPCR
P26: *LYZ*-RT-antisense	ATGCCTTGTGGATCACGGAC	RT-qPCR
P27: *xynB*-RT-sense	CGGCAGTGGAGGCACATACAAG	RT-qPCR
P28: *xynB*-RT-antisense	GGAAGCAGCATTGGTACGGGTAG	RT-qPCR

F, forward: R, reverse.

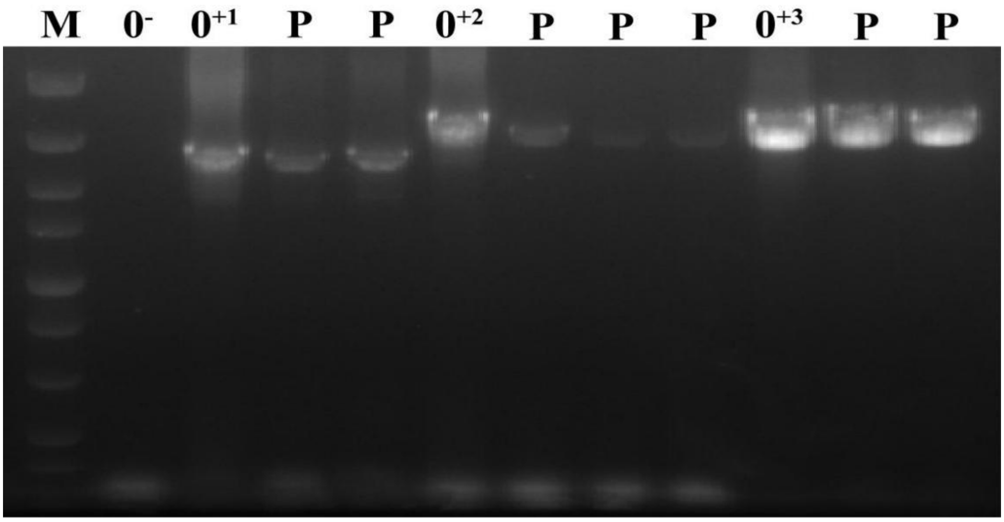

Supplementary Figure S1. Identification of inverters of *Agrobacterium* tumefaciens M represents marker; 0^- represents water (negative control); 0^{+1}, 0^{+2}, and 0^{+3} represent different recombinant plasmids (negative control); P stands for positive transformant.

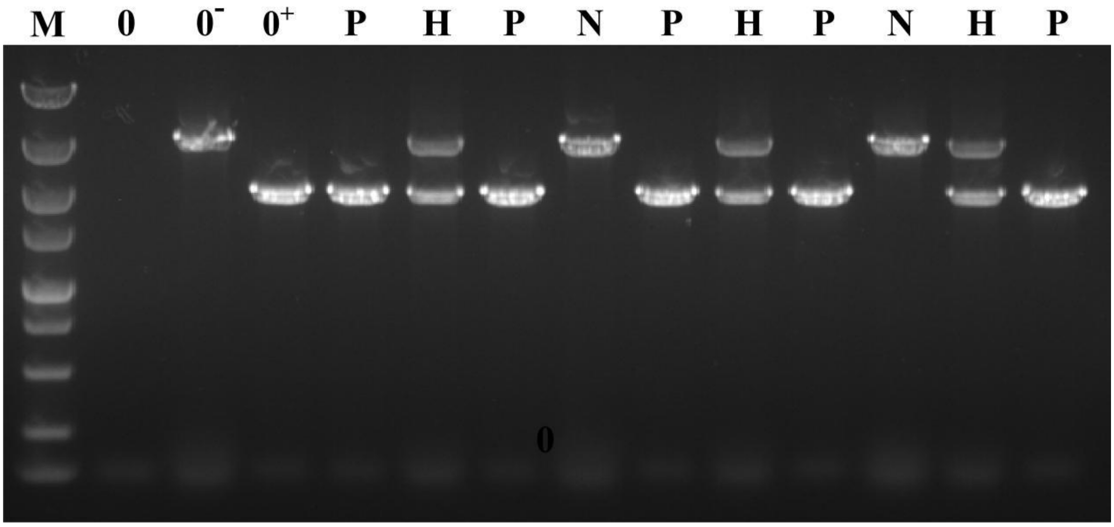

Supplementary Figure S2. Identification of inverters of *A. niger* M represents marker, 0 represents water, 0^- represents negative control (DNA of the originating strain TH-2), 0^+ represents positive control (recombinant plasmid); P stands for positive transformant; N stands for empty bacteria (negative transformant); H stands for heterozygous transformant.
